# Dépistage hospitalier de la drépanocytose en République Démocratique du Congo (RDC) par HemoTypeSC: cas de la ville de Kindu

**DOI:** 10.11604/pamj.2022.41.134.30187

**Published:** 2022-02-16

**Authors:** Abdala Kingwengwe Aimé, Shindano Mwamba Etienne, Destin Mbongi, Didier Nsonso, Erik Serrao, Tshilolo Muepu Malaika Léon, Luboya Numbi Oscar, Wembonyama Okitotsho Stanis

**Affiliations:** 1 Département de Pédiatrie, Faculté de Médecine, Université de Kindu, Kindu, République Démocratique du Congo,; 2 Unité de Prévention et Contrôle des Maladies, Faculté de Médecine, Université de Kindu, Kindu, République Démocratique du Congo,; 3 Département de Pédiatrie, Faculté de Médecine, Université de Lubumbashi, Lubumbashi, République Démocratique du Congo,; 4 Centre Hospitalier Mère-Enfant de Monkole, Kinshasa, République Démocratique du Congo,; 5 Silver Lake Research Corporation, Azusa, California, United States of America

**Keywords:** Drépanocytose, dépistage hospitalier, test rapide, diagnostic, HemoTypeSC, enfant, Kindu, République Démocratique du Congo, Sickle cell disease, hospital screening, rapid test, diagnostic, HemoTypeSC, child, Kindu, Democratic Republic of Congo

## Abstract

Le dépistage précoce de la drépanocytose est crucial pour améliorer la survie des personnes. L´accessibilité tant financière que géographique aux moyens diagnostiques de la drépanocytose est un obstacle au dépistage universel dans les pays en développement. Dans cette étude, nous avons déterminé la prévalence de la drépanocytose en milieu hospitalier chez les enfants de moins de 5 ans et avons évalué la validité de HemoTypeSC comme moyen de dépistage de la maladie dans un milieu aux conditions limitées. Il s´est agi d´une étude transversale et analytique à récolte prospective conduite au niveau des services de pédiatrie de 5 formations sanitaires de la Ville de Kindu, province du Maniema en RDC pendant 10 mois. L´étude a consisté en un dépistage de la drépanocytose à l´aide du test rapide HemoType SC puis une confirmation diagnostique par électrophorèse de l´hémoglobine. Au total 448 enfants de moins de 5 ans ont participé à l´étude. La prévalence hospitalière globale de la drépanocytose était de 31,9% dont 12,7% d´homozygotes SS et 19,2% des porteurs du trait; le niveau de suspicion de la drépanocytose en milieu hospitalier est de 6%; la présomption clinique de la drépanocytose est sensible à 8%; le test rapide HemoType SC présente des bons indicateurs de validité pour la détection des hémoglobines A et S. L´étude montre que le niveau de suspicion clinique de la drépanocytose est faible pour une région située dans la zone sicklémique. Cette suspicion doit être renforcée par le développement d´un profil clinique plus sensible permettant de justifier un dépistage ciblé. Aussi, le test rapide HemoTypeSC apparait fiable pour le dépistage de la maladie dans notre milieu.

## Abstract (en)

Early detection of sickle cell disease is crucial to improve people’s survival. Both financial and geographic accessibility to sickle cell disease tools are barriers to universal screening in developing countries. The purpose of this study was to determine the hospital prevalence of sickle cell disease and to assess the reliability of a rapid diagnostic tool, HemoTypeSC, in a resource-limited environment. We conducted a prospective cross-sectional descriptive study in the Department of Pediatrics of 5 health facilities in the city of Kindu, Maniema province, DRC, over a period of 10 months. The study consisted of HemoType SC rapid test screening for sickle cell disease and then diagnostic confirmation by hemoglobin electrophoresis. A total of 448 children less than 5 years of age were enrolled in the study. The overall hospital prevalence of patients with sickle cell disease was 31.9%, of whom 12.7% were homozygous (SS) and 19.2% trait carriers; the level of suspicion for sickle cell disease in hospitals was 6%; the clinical presumption regarding sickle cell disease was 8%; HemoType SC rapid test had good indicators of validity for the detection of hemoglobins A and S. The study shows that the hospital prevalence of major sickle cell disease is higher in children under 5 years of age with respect to clinical suspicion in the absence of laboratory tests. HemoTypeSC rapid test seems to be a reliable tool for the screening of the disease in the city of Kindu, a resource-limited environment.

## Introduction

Le dépistage vise à détecter les personnes à risque plus élevé d´une maladie ou un problème de santé au sein d´une population apparemment en bonne santé. Il permet à ce qu´un traitement ou une intervention précoce puissent leur être proposés, réduisant de ce fait l´incidence et/ou la mortalité due à cette maladie ou à ce problème de santé dans la dite population [1].

Hémoglobinopathie génétique la plus fréquente, la drépanocytose s´affiche parmi les problèmes de santé publique pour lesquels des actions sérieuses sont planifiées et menées [2-4]. Elle affecte majoritairement les populations des pays à faible revenu [5]. L´Afrique regorge près de 75% de toutes les personnes affectées par la maladie dans le monde [3,6].

La stratégie de lutte contre la maladie se fonde sur 3 actions : le dépistage, la bonne prise en charge et le conseil génétique [2,3,7,8]. Le succès de deux dernières actions est étroitement dépendant de la mise en œuvre effective d´un programme de dépistage. Le dépistage de la drépanocytose est idéalement souhaité à la naissance ou avant l´âge de 5 ans. Le manque d´un dépistage précoce occasionne le décès d´un grand nombre d´enfants drépanocytaires avant leur 5^e^ anniversaire [2,9,10].

Dans les pays industrialisés, le dépistage de la drépanocytose est devenu systématique sur base d´une orientation ciblée des populations à risque c´est-à-dire ayant un lien de filiation avec les originaires des foyers originels de la maladie [5,11,12]. Ces foyers sont la péninsule arabo-indienne, le bassin méditerranéen et l´Afrique intertropicale. Dans les pays à faible revenu, le dépistage n´est encore réalisé que dans les cadres des études ponctuelles [3,13,14].

Dans notre milieu, comme dans nombreux milieux en développement, la mise en œuvre d´un dépistage systématique se heurte à nombreuses difficultés entre autre la répartition inégale des outils diagnostics standards concentrés dans les grandes agglomérations et couteux [5,13]. Devant cette difficulté, il est impérieux de recourir aux actions alternatives pour accroitre le niveau du dépistage de la drépanocytose. Ces actions peuvent englober le recours aux moyens de dépistage moins couteux et simples pouvant-être utilisés en milieu hospitalier pédiatrique sans beaucoup de peines. Pour rationaliser les dépenses liées aux soins en milieu moins nanti, l´accroissement du niveau de suspicion clinique de la maladie peut représenter l´autre action complémentaire du dépistage.

L´objet de cette étude a été de déterminer la prévalence de la drépanocytose en milieu hospitalier parmi les enfants de moins de 5 ans et évaluer la validité des moyens diagnostiques applicables dans un milieu aux conditions limitées.

## Méthodes

Nous avons effectué une étude transversale, analytique à récolte prospective au niveau des services de pédiatrie de 5 formations sanitaires de la Ville de Kindu à savoir l´Hôpital Provincial de Référence de Kindu, de l´Hôpital Général de Référence d´Alunguli, du Centre Hospitalier Kitulizo, du Centre Hospitalier Lumbulumbu et du Centre de Santé de Référence CEPAC-Brazza. La Ville de Kindu est la capitale de la province du Maniema en République Démocratique du Congo. L´étude s´est déroulée du 02 décembre 2019 au 15 octobre 2020 soit 10 mois.

Les principaux critères d´inclusion ont été un délai transfusionnel de plus 120 jours avant l´enquête, un âge inférieur à 5 ans révolus et un consentement éclairé du tuteur. Le dépistage a été réalisé au moyen du test rapide HemoTypeSC et la confirmation de résultats a été faite par l´électrophorèse de l´hémoglobine (Hb) réalisée au laboratoire du Centre Hospitalier Mère-Enfant de Monkole à Kinshasa. L´électrophorèse de l´hémoglobine a servi de gold standard. Le statut drépanocytaire, dans cette étude, englobe le port du trait (AS ou AC) et la forme majeure (SS ou SC). Les sujets normaux sont ceux avec profil éléctrophorétique AA de l´hémoglobine.

HemoTypeSC est un test de flux latéral compétitif incorporant des anticorps monoclonaux pour la détection de l'hémoglobine A, de l'hémoglobine S et de l'hémoglobine C. Il permet de déterminer, à partir du sang total, les phénotypes de l'hémoglobine Hb AA (normal), Hb SS (drépanocytose homozygote S) et Hb CC (drépanocytose homozygote C), Hb SC (drépanocytose hétérozygote composite), Hb AS et Hb AC (trait drépanocytaire ou drépanocytose hétérozygote).

**Figure 1:**
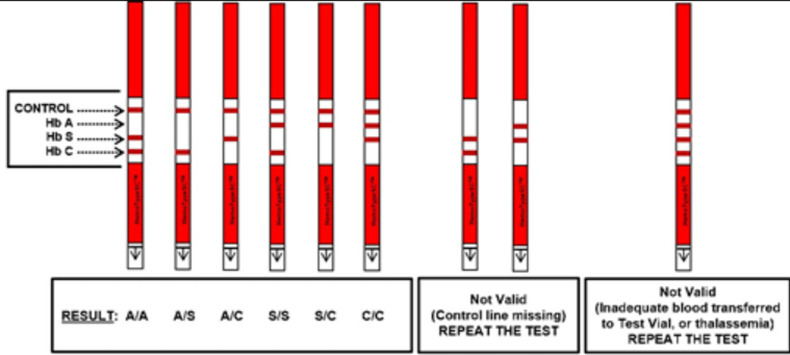
phénotypes d´hémoglobine détectée par test rapide HemoTypeSC [15]

L´interprétation du test est différente de nombreux autres tests rapides par le fait que pour HemoTypeSC, la présence d'une ligne indique l´absence du type d'hémoglobine correspondante. C´est par contre l´absence d'une ligne qui indique la présence du type d'hémoglobine (Figure 1) [15].

La présente étude a été conduite dans le respect des règles éthiques tant nationales qu´internationales. Elle a reçue préalablement un avis favorable du Comité d´éthique médicale de l´Université de Lubumbashi par sa lettre N° UNILU/CEM/023/2019.

## Résultats

Au total 448 enfants de moins de 5 ans dont 192 filles et 256 garçons ont participé à l´étude après consentement éclairé de leurs tuteurs.

Il apparait dans le Tableau 1 que la prévalence hospitalière globale de la drépanocytose chez les enfants de moins de 5 ans est de 31,9%, dont 12,7% de la forme homozygote SS et 19,2% de la forme hétérozygote. Dans la forme hétérozygote, nous avons noté la présence d´un cas porteur du trait C qui a représenté 0,2%.

**Tableau 1:** répartition des enquêtés selon leur profil éléctrophorétique de l´hémoglobine

Profil éléctrophorétique de l´Hb	Effectif	Pourcentage
AA	305	68,1
AS	85	19,0
AC	1	0,2
SS	57	12,7
**Total**	**448**	**100,0**

Il s´observe dans le Tableau 2 que la présomption clinique de la drépanocytose en milieu hospitalier est de 6% avec une indépendance par rapport aux résultats obtenus par électrophorèse de l´hémoglobine. Le test rapide HemoTypeSC a donné une prévalence drépanocytaire de 31,9% en milieu hospitalier avec une dépendance par rapport aux résultats obtenus par électrophorèse de l´hémoglobine.

**Tableau 2:** prévalence de détection de la drépanocytose par présomption clinique et HemoTypeSC

Méthodes diagnostiques	Drépanocytaires	Sujet normaux	p-value
Présomption clinique	27 (6,0%)	421	0,31
HemoTypeSC	143 (31,9%)	305	0,0001

Le Tableau 3 montre que la présomption clinique de la drépanocytose en milieu hospitalier est sensible à 8%. Par contre, la spécificité de la présomption clinique est de 95% avec des valeurs prédictives positive et négative respectivement de 41% et 69%.

**Tableau 3:** validité des méthodes diagnostiques du statut drépanocytaire par présomption clinique et HemoTypeSC

Statistiques	Présomption clinique	IC à 95%	HemoTypeSC	IC à 95%
Sensibilité (%)	8	(4,3-13,4)	100	(96,8-100,0)
Spécificité (%)	95	(91,6-96,8)	100	(98,5-100,0)
VPP (%)	41	(22,2-59,3)	100	(100,0-100,0)
VPN (%)	69	(64,2-71,1)	100	(100,0-100,0)

À l´opposé de la présomption clinique, le Tableau 3 indique que HemoTypeSC a une sensibilité de 100%, une spécificité de 100% avec des valeurs prédictives positive et négative de 100% chacune.

À la lecture du Tableau 4, nous constatons que le test rapide HemoTypeSC présente des bons indicateurs de validité en ce qui concerne la détection des hémoglobines A et S. Les indicateurs de validité du test rapide HemoTypeSC affichent des résultats mitigés en ce qui concerne l´hémoglobine C.

**Tableau 4:** validité du test rapide HemoTypeSC pour la détection de chaque type d´hémoglobine (Hb)

Type d´Hb	Sensibilité (%)	Spécificité (%)	VPP (%)	VPN (%)
Hb A	100 (IC : 98,8-100,0)	100 (IC : 92,3-100,0)	100 (IC : 100,0-100,0)	100 (IC : 100,0-100,0)
Hb S	100 (IC : 96,7-100,0)	99,7 (IC : 97,9-100,0)	99,3 (IC : 97,9-100,0)	100 (IC : 100,0-100,0)
Hb C	0,0 (IC : 0,0-82,9)	100 (IC : 98,9-100,0)	Indéterminée	99,8 (IC : 99,3-100,0)

## Discussion

Notre étude a relevé une prévalence hospitalière de la drépanocytose à 31,9% dont 12,7% de la forme homozygote SS et 19,2% de la forme hétérozygote auprès des enfants de moins de 5 ans. Dans la littérature, une étude presque similaire à la nôtre et menée au Soudan a trouvé une prévalence hospitalière de la drépanocytose à 14,8% dont 3,5% de la forme homozygote SS et 11,3% de la forme hétérozygote auprès d'enfants de 0 à 18 ans admis à l'hôpital universitaire Al Fashir. Toutefois, nos résultats sont supérieurs à ceux de nombreuses études qui ont déterminé la fréquence hospitalière de la drépanocytose sans que cela ne soit le fruit d´un dépistage. C´est le cas de Elie *et al*. [16] au Togo, Keita [17] et Thiero [18] au Mali qui ont trouvé des fréquences respectives de 3,2%, 1,5% et 1,3% d´enfants admis avec syndrome drépanocytaire majeur alors que dans notre série leur fréquence atteint 12,7%. L´OMS, à partir d´une étude menée en RD Congo en 2009 estime que 12% d´enfants admis dans les services de pédiatrie sont drépanocytaires sans faire la distinction entre les porteurs du trait et ceux avec la forme majeure [2,19]. Nos résultats démontrent la sous-estimation de la prévalence de la drépanocytose en milieu hospitalier en l´absence d´un dépistage.

La suspicion clinique de la drépanocytose dans notre milieu est de l´ordre de 6% dans la population infanto-juvénile. Il n´existe pas, à notre connaissance, un niveau de suspicion de la drépanocytose qui soit recommandé. Toutefois, le chiffre obtenu dans notre série nous parait bas pour un milieu situé dans la région sicklémique et la proportion de la drépanocytose révélée par notre enquête. Ce bas niveau de suspicion de la maladie pourrait être lié à deux facteurs; un faible niveau de connaissance des prestataires des soins sur le diagnostic de la maladie [20] et l´absence d´un outil d´orientation clinique contextualisé. Pour pouvoir accroître cette suspicion, il faut renforcer les connaissances des prestataires des soins sur le diagnostic de la drépanocytose et proposer un profil contextualisé pour la suspicion de la maladie au sein de la population infanto-juvénile hospitalière.

La présomption clinique de la drépanocytose dans notre série présente une sensibilité de 8%, une spécificité de 95% avec des valeurs prédictives positive et négative respectivement de 41% et 69%. Nos résultats montrent que la présomption clinique existante n´est pas statistiquement valide pour orienter la recherche des cas de drépanocytose dans la population infanto-juvénile. Pour parvenir à optimiser la recherche des cas de la maladie, la présomption clinique doit-être la plus sensible que possible [1]. Dans notre série, toutes les statistiques de validité du test rapide HemoTypeSC sont à 100% pour la détection du statut drépanocytaire. Ces résultats corroborent ceux présentés par les études de Quinn CT [5], Steele C [21], Nankanja *et al*. [22] et Kasai *et al*. [23]. Le test rapide HemoTypeSC peut donc être recommandé comme moyen de dépistage fiable dans notre milieu en complément de la suspicion clinique.

S´agissant de la détection des types d´hémoglobine, HemoTypeSC apparait tant sensible que spécifique pour les hémoglobines A et S. Par contre ses indicateurs de validité dans la détection de l´hémoglobine C sont sans conclusion. Quinn [24] et Steele [21] ont trouvé des résultats similaires aux nôtres en ce qui concernent la validité du test rapide HemoTypeSC dans la détection des hémoglobines A et S. il s´observe une différence des résultats entre ces auteurs et nous quant à ce qui concerne la détection de l´hémoglobine C. La faible prévalence de l´hémoglobine C dans notre échantillon pourrait expliquer l´indétermination des indicateurs de validité de HemoTypeSC. La prévalence d´un phénomène influence le niveau de validité des tests diagnostiques lui destinés [1].

## Conclusion

L´étude montre que le niveau de suspicion clinique de la drépanocytose est fiable pour une région située dans la zone sicklémique. Aussi, la prévalence hospitalière de la drépanocytose est élevée dans notre milieu au sein de la population infanto-juvénile. Il y a lieu d´accroître cette suspicion par l´élaboration d´un profil orientatif pour ainsi justifier un dépistage ciblé. Aussi, le test rapide HemoTypeSC apparait valide pour le dépistage de la maladie dans notre milieu. Ceci ne doit pas exclure l´obligation d´un deuxième test de confirmation.

### Etat des connaissances sur le sujet

- Données parcellaires sur la drépanocytose;
- Accessibilité difficile aux outils diagnostiques.

### Contribution de notre étude à la connaissance

- Prevalence hospitalière infanto-juvénile concrète basée sur un dépistage systématique;
- Fiabilité d´un test rapide utilisable dans les conditions limitées.

## Remerciements

Equipe ayant aidé au recrutement des enquêtés. Les tuteurs ayant consenti pour la participation des enfants à l´enquête. Le Centre Hospitalier Mère-Enfant de Monkole pour l´analyse des échantillons à l´électrophorèse.
